# Antenatal Corticosteroid Treatment During the Late-Preterm Period and Neonatal Outcomes for Twin Pregnancies

**DOI:** 10.1001/jamanetworkopen.2023.43781

**Published:** 2023-11-17

**Authors:** Jie Zhu, Ying Zhao, Ping An, Yunhe Zhao, Shuyue Li, Jizi Zhou, Huanqiang Zhao, Qiongjie Zhou, Xiaotian Li, Yu Xiong

**Affiliations:** 1 Department of Obstetrics, Obstetrics and Gynecology Hospital of Fudan University, Shanghai, China; 2 Department of Obstetrics, Shenzhen Maternity and Child Healthcare Hospital, Shenzhen, Guangdong, China

## Abstract

**Importance:**

Antenatal corticosteroid treatment of individuals with singletons at risk for delivery during the late-preterm period has been academically recommended. However, the evidence on the use of antenatal corticosteroid treatment for twins at risk for delivery during the late-preterm period is still lacking.

**Objective:**

To evaluate whether antenatal corticosteroid treatment during the late-preterm period in twin pregnancies was associated with a lower risk of newborn morbidity.

**Design, Setting, and Participants:**

This retrospective cohort study of twin pregnancies delivered from February 1, 2013, to September 30, 2020, in a university-affiliated hospital in China included 1974 individuals with twin pregnancies who were at risk for late preterm birth (34 weeks and 0 days to 36 weeks and 6 days of gestation). Data were analyzed from June 30 to July 13, 2023.

**Exposures:**

Antenatal corticosteroid treatment during the late-preterm period.

**Main Outcomes and Measures:**

The primary outcome measure was composite neonatal respiratory morbidity, defined as at least 1 of the following postnatal occurrences in at least 1 neonate of the twins: respiratory distress syndrome, mechanical ventilation, surfactant administration, transferred with respiratory complications, or neonatal death. Propensity score overlap weighting was used to analyze the association between antenatal corticosteroid treatment and the risk of neonatal outcomes.

**Results:**

The study population consisted of 1974 individuals with twin pregnancies, including 303 (15.3%; mean [SD] maternal age, 30.8 [4.2] years) who received antenatal corticosteroid treatment and 1671 (84.7%; mean [SD] maternal age, 31.2 [4.0] years) who did not receive antenatal corticosteroid treatment. The propensity score overlap weighting showed no significant differences between the antenatal corticosteroid treatment group and the no–antenatal corticosteroid treatment group in the risk of neonatal primary outcome (29 of 303 [9.6%] vs 41 of 1671 [2.5%]; weighted odds ratio, 1.27 [95% CI, 0.60-2.76]). None of the subgroup interaction tests were significant for the neonatal primary outcome in terms of gestational age at delivery, year of delivery, chorionicity, at least 1 infant small for gestational age, intertwin growth discordance, and infant sex, and neither was the sensitivity analysis of using propensity score matching and a different administration-to-birth interval and treating twin infants as individuals.

**Conclusions and Relevance:**

This cohort study found insufficient evidence that antenatal corticosteroid treatment during the late-preterm period in twin pregnancies could be associated with a lower risk of newborn morbidity. This new finding can provide a reference for clinical practice.

## Key Points

**Question:**

Is there evidence for an association of antenatal corticosteroid treatment during the late-preterm period with an improvement in twins’ neonatal outcomes?

**Findings:**

In this cohort study, antenatal corticosteroid treatment of 1974 individuals with twin pregnancies and at risk for late preterm birth did not significantly reduce the risk of neonatal respiratory morbidity as well as other neonatal complications.

**Meaning:**

The evidence is insufficient that antenatal corticosteroid treatment during the late-preterm period in twin pregnancies is associated with a lower risk of newborn morbidity.

## Teaser

This cohort study evaluates whether antenatal corticosteroid treatment during the late-preterm period in twin pregnancies is associated with a lower risk of newborn morbidity.

## Introduction

Over the past 4 decades, the prevalence of twin pregnancies has increased substantially and globally.^1,2^ Meanwhile, the incidence of preterm births for twin pregnancies has remained notably high.^3,4^ Among twins, those born in the late-preterm period (34 weeks and 0 days to 36 weeks and 6 days of gestation) comprise approximately 70% of twin pregnancies and experience a significantly higher rate of respiratory complications than do full-term infants.^5,6,7^

Antenatal corticosteroids are generally administered to pregnant women at risk for premature delivery between 24 weeks and 0 days and 33 weeks and 6 days of gestation during both singleton and twin pregnancies to accelerate fetal lung maturation and decrease the incidence of neonatal mortality and morbidity.^8^ After the Antenatal Late Preterm Steroids (ALPS) randomized clinical trial was completed and published in 2016, the American College of Obstetricians and Gynecologists (ACOG) and Society for Maternal-Fetal Medicine (SMFM) endorsed the use of antenatal corticosteroids (2 doses of betamethasone) for pregnant women at risk of imminent birth in the late-preterm period, on the basis of the previously reported findings among singletons.^9,10,11^ As for twins in the late-preterm period, however, the evidence was insufficient to guide clinical practice. Only a few cohort studies, which were characterized by small sample sizes, unmatched comparisons, and great heterogeneity, have been reported. The results showed that the use of antenatal corticosteroid treatment was not consistently associated with an improving neonatal respiratory outcome.^12,13^ Therefore, the correlation between antenatal corticosteroid treatment for twins at risk of late-preterm birth and neonatal outcomes remains an unanswered question.

To better assess the association between antenatal corticosteroid treatment during the late-preterm period and neonatal outcomes of twin pregnancies, we conducted a retrospective cohort study of 2705 twin pregnancies. We used propensity score overlap weighting to make the maternal and neonatal baseline information comparable. Furthermore, to ensure the robustness of the findings, we performed subgroup analysis on potential interactions with the specific variables, including chorionicity, at least 1 infant small for gestational age (SGA), intertwin growth discordance, and infant sex, and we performed sensitivity analysis using propensity score matching and a different administration-to-birth interval and treating twin infants as individuals.

## Methods

### Study Participants

The retrospective cohort involved all individuals whose twin pregnancies were delivered from February 1, 2013, to September 30, 2020, at the Obstetrics and Gynecology Hospital of Fudan University, Shanghai, China, a tertiary hospital. The individuals had been identified from the institution’s electronic medical record system, and their electronic records were extracted for the relevant data on maternal characteristics, pregnancy outcomes, neonatal outcomes, and antenatal corticosteroid treatment. Gestational age (GA) was calculated based on the last menstrual period, adjusted by the crown-rump length in the first trimester or the head circumference of the larger fetus with fetal ultrasonographic examination after 14 weeks of gestation in the case of spontaneous conception, as well as based on the timing of in vitro pregnancy fertilization conceived via assisted reproductive technology. Our study conformed to the Strengthening the Reporting of Observational Studies in Epidemiology (STROBE) reporting guideline for cohort studies. The study was approved by the Ethics Committee of Obstetrics and Gynecology Hospital of Fudan University, and the requirement for informed consent was waived because the data were deidentified.

**Figure 1.**
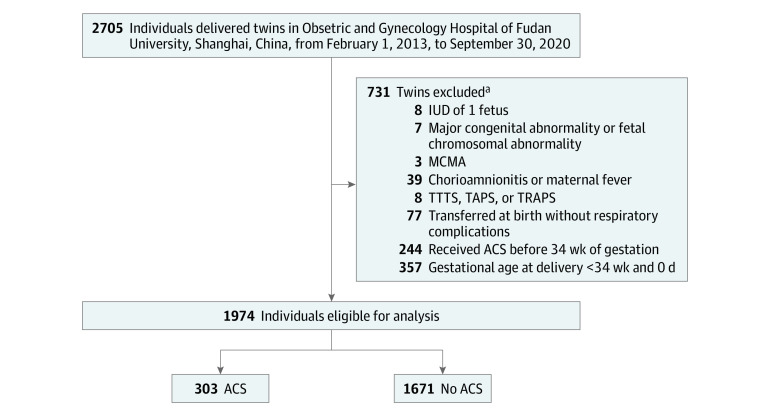
Description of the Study Cohort ACS indicates antenatal corticosteroid; IUD, intrauterine death; MCMA, monochorionic monoamniotic; TAPS, twin anemia-polycythemia sequence; TRAPS, twin reversed arterial perfusion sequence; and TTTS, twin-to-twin transfusion syndrome. ^a^Individuals could fulfill more than 1 exclusion criterion.

The inclusion criteria were as follows: (1) establishment of a prenatal medical record at our hospital and (2) receipt of the routine prenatal care offered by our hospital until delivery. The exclusion criteria were as follows: (1) GA of less than 34 weeks at delivery; (2) intrauterine death of 1 fetus; (3) major congenital abnormality or fetal chromosomal abnormality; (4) monochorionic monoamniotic twins; (5) chorioamnionitis or maternal fever; (6) complications unique to monochorionic twins, including twin-to-twin transfusion syndrome, twin reversed arterial perfusion sequence, or twin anemia-polycythemia sequence; (7) missing outcomes because of being transferred at birth without respiratory complications; or (8) antenatal corticosteroid treatment received before 34 weeks and 0 days of gestation (Figure 1).

### Exposure

The exposure was set as follows: at least 1 dose of antenatal corticosteroids was administered between 34 weeks and 0 days and 36 weeks and 6 days of gestation, in view of a high probability of delivery in the late-preterm period. The protocol for antenatal corticosteroid treatment comprises 4 intramuscular doses of 5 mg of dexamethasone 12 hours apart.^14,15^ The high probability of delivery was defined as the spontaneous rupture of the membranes, intact membranes and at least 3 cm of dilation or 75% cervical effacement, or a preterm delivery expected for any other indication for which the attending physician decides to induce labor or perform cesarean delivery within 24 hours and 7 days. After birth, maternal characteristics and neonatal outcomes were compared between twins in 2 study groups: those who received at least 1 dose of dexamethasone from 34 weeks and 0 days to 36 weeks and 6 days of gestation and those who did not.

### Outcomes

The primary outcome measure was composite neonatal respiratory morbidity, defined as at least 1 of the following postnatal occurrences to either neonate: respiratory distress syndrome (at delivery), mechanical ventilation (within 72 hours of life), surfactant administration (at delivery), transferred with respiratory complications (at delivery), or neonatal death (within 72 hours of life). Transferred with respiratory complications was defined as having a poor respiratory condition, such as respiratory distress syndrome, for which the neonate was transferred to the local children’s hospital for further evaluation and treatment by neonatologists.

The secondary outcomes were referred to as the use of continuous positive airway pressure (CPAP), the use of CPAP for more than 2 consecutive days, asphyxia, severe asphyxia, transient tachypnea, admission to the neonatal intensive care unit for any duration or for no less than 3 consecutive days, necrotizing enterocolitis, sepsis, hyperbilirubinemia, hyperglycemia, hypoglycemia, and acidosis. Hypoglycemia was defined as a glucose level of less than 40 mg/dL (to convert to millimoles per liter, multiply by 0.0555) tested at any time. The definitions of variables and outcomes are provided in eTable 1 in Supplement 1.

### Statistical Analysis

Data were analyzed from June 30 to July 13, 2023. The baseline characteristics of the 2 study groups were compared. Continuous variables with normal distributions were presented as mean (SD) values, and categorical data were presented as numbers and percentages. The *F* test was performed for the comparison of quantitative data, and the χ^2^ test or the Fisher exact test was performed for the comparison of categorical data.

For adjusting between-group differences, propensity scores were developed to reflect the probability of each participant receiving antenatal corticosteroid treatment, with the use of multivariable logistic regression with exposure (antenatal corticosteroid treatment vs no antenatal corticosteroid treatment) as the dependent variable and all observed maternal-level and neonatal-level characteristics as the independent variables. The covariates covered baseline maternal sociodemographic and clinical factors, as well as neonatal characteristics, which were expected to be associated with neonatal outcomes. These covariates included year of delivery (2013-2016 and 2017-2020, as the SMFM and the ACOG altered their guidelines regarding antenatal corticosteroid treatment in 2016 to include individuals at risk for late-preterm delivery^9,10^), advanced maternal age (≥35 years at delivery), primipara, assisted reproductive technology, gestational diabetes, hypertensive disorders during pregnancy, GA at delivery, preterm birth, mode of delivery (vaginal or cesarean), chorionicity (monochorionicity or dichorionicity), mean birth weight of both twin infants from the same mother, at least 1 infant SGA, intertwin growth discordance, and infant sex (the same or opposite).

Based on propensity scores, 4 adjustment approaches were used to match the baseline of 2 groups, including inverse probability of treatment weighting, standardized mortality ratio weighting, overlap weighting, and propensity score matching (PSM). Inverse probability of treatment weighting, standardized mortality ratio weighting, and overlap weighting, as well as the approach of PSM of 1 to 1, 2, 3, and 4 matching patients with the nearest neighbor matching algorithm (a caliper width of 0.1), were performed to examine the association between antenatal corticosteroid treatment and neonatal outcomes. Standardized mean differences were used to select the optimal adjustment method. As the absolute standardized mean difference of all covariates was smaller than 10% to support the assumption of balance between study groups,^16^ overlap weighting was chosen for subsequent research (eFigure 1 in Supplement 1). The outcomes were investigated using univariate logistic regression. Odds ratios (ORs) with 95% CIs were used to show the effect size of a comparison.

We conducted 6 subgroup analyses of the primary outcome to examine the heterogeneity in clinically relevant subgroups: GA at delivery (34 weeks and 0 days to 34 weeks and 6 days, 35 weeks and 0 to 35 weeks and 6 days, and 36 weeks and 0 days to 36 weeks and 6 days), year of delivery, chorionicity, at least 1 infant with SGA, intertwin growth discordance, and infant sex. The robustness of the results was examined via multiple sensitivity analyses. First, we performed 1:1 PSM as an alternative to overlap weighting because the absolute standardized mean difference of almost all covariates was smaller than 10% (eFigure 1 in Supplement 1). Second, we regarded twin neonates as individuals, adding birth order, infant sex, and birth weight to the covariates. In addition, we further divided the antenatal corticosteroid treatment group into 3 groups based on the time from the administration of the first dose of dexamethasone to birth—less than 24 hours, 24 hours to 7 days, and more than 7 days—and used multivariate logistic regression analyses to assess whether the administration-to-birth interval was a factor in the association of antenatal corticosteroid treatment with neonatal outcomes.

A 2-tailed *P* < .05 was considered statistically significant. All statistical analyses were conducted with R statistical software, version 4.2.2 (R Project for Statistical Computing).

## Results

A total of 2705 individuals with twin pregnancies were eligible for the current retrospective analysis, of whom 731 met the exclusion criteria. Thus, the study population comprised 1974 individuals with twin pregnancies: 303 (15.3%; mean [SD] maternal age, 30.8 [4.2] years) who received antenatal corticosteroid treatment between 34 weeks and 0 days to 36 weeks and 6 days of gestation and 1671 (84.7%; mean [SD] maternal age, 31.2 [4.0] years) who did not receive antenatal corticosteroid treatment (Figure 1 and Table 1).

**Table 1.** Baseline Characteristics of the Study Cohorts

Characteristic	No-ACS group, No. (%) (n = 1671)	ACS group, No. (%) (n = 303)	*P* value	Absolute standardized mean difference
Maternal characteristics				
Year of delivery				
2013-2016	931 (55.7)	199 (65.7)	.001	0.205
2017-2020	740 (44.3)	104 (34.3)		
Maternal age, mean (SD), y	31.2 (4.0)	30.8 (4.2)	.08	0.108
Advanced maternal age	335 (20.0)	57 (18.8)	.70	0.031
Primipara	1537 (92.0)	267 (88.1)	.03	0.129
ART	899 (53.8)	138 (45.5)	.009	0.166
GD	332 (19.9)	61 (20.1)	.94	0.007
HDP	395 (23.6)	103 (34.0)	<.001	0.230
Gestational age, wk				
34	80 (4.8)	78 (25.7)	<.001	1.236
35	177 (10.6)	79 (26.1)		
36	454 (27.2)	114 (37.6)		
37	815 (48.8)	30 (9.9)		
38	135 (8.1)	2 (0.7)		
39	4 (0.2)	0		
40	6 (0.4)	0		
Preterm birth	711 (42.5)	271 (89.4)	<.001	1.139
Indication for preterm birth, No./total No. (%)				
Preterm labor with intact membranes	43/711 (6.0)	15/271 (5.5)	.07	0.219
Ruptured membranes	151/711 (21.2)	63/271 (23.2)		
Expected delivery for HDP	186/711 (26.2)	90/271 (33.2)		
Expected delivery for FGR	30/711 (4.2)	5/271 (1.8)		
Expected delivery for other indication^a^	301/711 (42.3)	98/271 (36.2)		
Cesarean delivery	66 (3.9)	9 (3.0)	.51	0.054
Dichorionicity	1379 (82.5)	201 (66.3)	<.001	0.378
Neonatal characteristics				
Birth weight of both twin infants from same mother, mean (SD), g	2610.6 (289.8)	2371.5 (328.3)	<.001	0.772
At least 1 infant with SGA	589 (35.2)	86 (28.4)	.02	0.148
Growth discordance	203 (12.1)	43 (14.2)	.34	0.060
Same-sex twin	1056 (63.2)	216 (71.3)	.007	0.173

Abbreviations: ACS, antenatal corticosteroid; ART, assisted reproductive technology; FGR, fetal growth restriction; GD, gestational diabetes; HDP, hypertensive disorders in pregnancy; SGA, small for gestational age.

^a^ Other indications: breech presentation, shoulder presentation, placenta accrete, placenta previa, intrahepatic cholestasis of pregnancy, monochorionic twins, or others.

A total of 271 individuals (89.4%) in the antenatal corticosteroid treatment group delivered before 37 weeks and 0 days of gestation, while only 711 individuals (42.5%) in the no–antenatal corticosteroid treatment group did (*P* < .001). Also, several baseline characteristics were significantly different between groups, with the antenatal corticosteroid treatment group having a lower mean (SD) birth weight than the no–antenatal corticosteroid treatment group (2371.5 [328.3] vs 2610.6 [289.8] g; *P* < .001) (Table 1).

**Figure 2.**
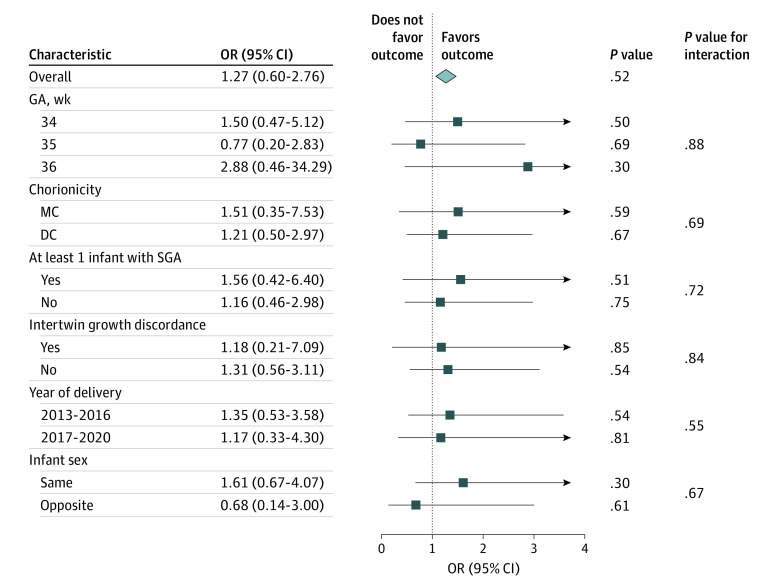
Association of Antenatal Corticosteroids With Primary Neonatal Outcomes in the Prespecified Subgroups After Overlap Weighting DC indicates dichorionicity; GA, gestational age; MC, monochorionicity; OR, odds ratio; and SGA, small for gestational age.

After overlap weighting, the univariate logistic regression analysis showed no significant differences between the antenatal corticosteroid treatment group and the no–antenatal corticosteroid treatment group in the risk of neonatal primary outcome (29 of 303 [9.6%] vs 41 of 1671 [2.5%]; weighted OR, 1.27 [95% CI, 0.60-2.76]). Similar results were shown for the risk of being transferred with respiratory complications, respiratory distress syndrome, and surfactant administration. The risks of other neonatal complications, such as CPAP use, CPAP use for more than 2 days, asphyxia, severe asphyxia, transient tachypnea, hyperbilirubinemia, hyperglycemia, hypoglycemia, and acidosis, also did not differ significantly (Table 2). Subgroup analysis showed no significant interaction for the neonatal primary outcomes between antenatal corticosteroid treatment and other factors, such as GA at delivery, year of delivery, chorionicity, at least 1 infant with SGA, intertwin growth discordance, and infant sex (Figure 2).

**Table 2.** Association of ACS Administration With Neonatal Outcomes

Outcome^a^	Crude (unweighted)		After overlap weighting			
	No-ACS group, No. (%) (n = 1671)	ACS group, No. (%) (n = 303)	No-ACS group, %	ACS group, %	OR (95% CI)	*P* value
Primary outcomes^b^	41 (2.5)	29 (9.6)	6.2	7.8	1.27 (0.60-2.76)	.52
Transferred with respiratory complications	16 (1.0)	12 (4.0)	2.1	3.5	1.71 (0.53-6.23)	.38
Mechanical ventilation	4 (0.2)	0	0.2	0	NA	NA
Respiratory distress syndrome	25 (1.5)	21 (6.9)	4.5	5.4	1.21 (0.50-3.01)	0.67
Surfactant use	28 (1.7)	21 (6.9)	4.8	5.5	1.16 (0.48-2.80)	0.74
Neonatal death	1 (0.1)	0	0	0	NA	NA
Secondary outcomes						
CPAP use	93 (5.6)	49 (16.2)	14.0	13.2	0.93 (0.53-1.63)	.81
CPAP use for >2 d	26 (1.6)	15 (5.0)	4.2	4.1	0.97 (0.37-2.56)	.95
Asphyxia	51 (3.1)	6 (2.0)	3.5	1.5	0.43 (0.10-1.50)	.21
Severe asphyxia	12 (0.7)	3 (1.0)	0.6	0.8	1.43 (0.12-24.71)	.77
Transient tachypnea	338 (20.2)	129 (42.6)	37.0	37.8	1.04 (0.70-1.54)	.86
NICU	805 (48.2)	225 (74.3)	69.1	69.2	1.01 (0.67-1.52)	.97
NICU for ≥3 d	763 (45.7)	212 (70.0)	65.5	65.3	0.99 (0.66-1.48)	.97
Necrotizing enterocolitis	1 (0.1)	0	0	0	NA	NA
Sepsis	6 (0.4)	0	0.8	0	NA	NA
Hyperbilirubinemia	397 (23.8)	95 (31.4)	36.3	29.4	0.73 (0.48-1.09)	.13
Hyperglycemia	24 (1.4)	7 (2.3)	3.5	2.2	0.62 (0.18-1.97)	.43
Hypoglycemia	69 (4.1)	33 (10.9)	7.1	10.1	1.47 (0.74-2.97)	.28
Acidosis	184 (11.0)	68 (22.4)	20.9	19.6	0.92 (0.57-1.48)	.74

Abbreviations: ACS, antenatal corticosteroid; CPAP, continuous positive airway pressure; NA, not applicable; NICU, neonatal intensive care unit; OR, odds ratio.

^a^ Definitions of outcome measures listed in eTable 1 in Supplement 1.

^b^ The primary outcome measure was a composite neonatal respiratory morbidity outcome defined as at least 1 of the following occurrences in at least 1 neonate of the twins: respiratory distress syndrome, mechanical ventilation, surfactant administration, transferred with respiratory complications, or neonatal death.

The sensitivity analyses showed a similar result in terms of the association of antenatal corticosteroid treatment with neonatal outcomes after 1:1 PSM (eTables 2 and 3 in Supplement 1) and after treating twin neonates as separate individuals (eTables 4 and 5 in Supplement 1). From the multivariable logistic regression analysis to investigate the association of the administration-to-birth interval with the association of antenatal corticosteroid treatment with neonatal outcomes, no differences were observed in the risk of neonatal primary outcomes at different intervals. As to the secondary neonatal outcomes, the risk of neonatal hypoglycemia was significantly higher in the antenatal corticosteroid treatment group than in the no–antenatal corticosteroid treatment group at the interval of 24 hours to 7 days (adjusted OR [aOR], 1.92 [95% CI, 1.29-2.81]), while the risks of neonatal hyperbilirubinemia (aOR, 0.60 [95% CI, 0.39-0.90]) and acidosis (aOR, 0.49 [95% CI, 0.49-0.84]) were significantly lower in the antenatal corticosteroid treatment group than in the no–antenatal corticosteroid treatment group at the interval of less than 24 hours and more than 7 days, respectively (eFigure 2 and eTables 6, 7, and 8 in Supplement 1).

## Discussion

In this large, single-center retrospective cohort study, we did not find evidence that antenatal corticosteroid treatment of individuals with twin pregnancy at risk for late-preterm birth could reduce the risk of neonatal respiratory morbidity. The finding was confirmed by the subgroup interaction tests, including GA at delivery, year of delivery, chorionicity, at least 1 infant with SGA, intertwin growth discordance, and infant sex, as well as sensitivity analysis using propensity score matching and a different administration-to-birth interval and treating twin infants as individuals. However, the risk of neonatal hypoglycemia increased significantly when antenatal corticosteroid treatment was administered 1 to 7 days prior to birth, as was previously reported.^17^

There has been considerable evidence supporting antenatal corticosteroid treatment during the late-preterm period for singleton pregnancies^11,18,19^ but limited, unsupportive evidence for antenatal corticosteroid treatment for twin pregnancies.^12,13^ This difference could be explained by the differences in the pharmacokinetics of corticosteroids in twins vs singletons, the off-target phenomenon of antenatal corticosteroids (the absence of delivery ≤7 days after administration of antenatal corticosteroids), or the confounding factors associated with antenatal corticosteroid choice.

To minimize the confounding factors in antenatal corticosteroid treatment of individuals with twin pregnancy, we implemented the following strategies. We took advantage of a cohort of 2705 individuals with twin pregnancies, which, to our knowledge, is larger than that of previously reported studies.^12,13^ Instead of the conventional multivariable logistic regression previous studies used, we used the propensity score overlap weighting method to effectively eliminate the potential confounding factors, achieving the effect of randomized clinical trials to the greatest extent possible in retrospective studies.^20^ For outcome measures, based on earlier studies, such as the ALPS trial and the clinical realities of our center, we developed outcome indicators that could comprehensively reflect the association between antenatal corticosteroid treatment and neonatal outcomes among twins.^11^ As there are no randomized clinical trials on the effect of antenatal corticosteroid treatment in twin pregnancy to our knowledge, we believe that carefully designed observational studies can be of great help in treatment decision-making. Next, the results did not change significantly in the multiple subgroup and sensitivity analyses, showing that the results were robust enough to be convincing and reliable.

As antenatal corticosteroids have shown the greatest benefit if the infant is delivered 24 to 48 hours after the initial dose and within 7 days of the administration, the off-target phenomenon of antenatal corticosteroid treatment refers to the absence of delivery within the 7 days after antenatal corticosteroid treatment.^21,22,23,24^ Unlike the 2 previously reported investigations,^12,13^ our study aimed to explore the potential association of the administration-to-birth interval with outcomes; we found that antenatal corticosteroid treatment, regardless of the length of the interval, was not associated with improved neonatal respiratory outcomes, which suggested that our findings were not associated with the off-target phenomenon.

The lack of benefit associated with antenatal corticosteroid treatment in twin pregnancies may be attributed to the different pharmacokinetics of corticosteroids between twins and singletons. Corticosteroids are thought to have a shorter half-life and a higher clearance rate among twins, and the physiological changes in the maternal blood volume during twin pregnancy may affect drug distribution, all of which may lead, for twins, to lower effective therapeutic doses of corticosteroids that are intended to promote fetal lung maturation.^25,26^ Although the beneficial association of corticosteroids with respiratory outcomes for early-preterm singleton infants is widely recognized, controversy still exists over their association with outcomes for early-preterm twin infants, with large-sample studies demonstrating no improvement in respiratory outcomes.^27,28^ Hence, the corticosteroid dosage currently in use is probably not optimal enough to promote fetal lung maturation in twin pregnancies.

Our study suggested that antenatal corticosteroid treatment for twin pregnancies at risk of late-preterm birth could not decrease neonatal respiratory morbidity but could increase the risk of neonatal hypoglycemia when administered 1 to 7 days prior to birth. Thus, antenatal corticosteroids should not be administered to those who do not have a high likelihood of preterm birth with twin pregnancies until more robust evidence is available. The current shortcomings in the use of antenatal corticosteroid treatment for twins should be properly addressed, such as the lack of data on optimal dosage, estimating preterm birth, and the long-term effect on multiple systems. Further randomized clinical trials are required to help determine optimal antenatal corticosteroid treatment recommendations for this special risk group of women.

### Limitations

This study has several limitations. First, this cohort study performed a retrospective observational analysis. Although we used propensity score overlap weighting to balance the differences between groups, unmeasured and residual confounding could still exist. Second, although one of the primary outcome indicators was patient transferred with respiratory complications, the information on the prognosis of these newborns was inevitably lost, which increased the potential for bias. Third, due to the challenges in estimating the timing of delivery, only approximately 30% of the antenatal corticosteroid treatment group completed the full course of corticosteroids, which may have led to bias in the outcomes. Fourth, the findings need to be interpreted with caution because some neonatal outcomes, such as death in the late-preterm period, had a low incidence. Fifth, the cohort study spanned approximately 8 years, during which the changes in clinical practice, medical standards, and policy guidelines could have exerted a potential effect on the generalizability of the conclusion.

## Conclusion

In this cohort study, evidence was insufficient that antenatal corticosteroid treatment for a twin pregnancy during the late-preterm period could be associated with a lower risk of newborn morbidity. However, antenatal corticosteroid treatment 1 to 7 days before birth was associated with a higher risk of neonatal hypoglycemia. More well-designed prospective studies are required.

## Notes

Supplement 1. **eTable 1.** Definitions of Variables and Outcome Measures **eFigure 1.** Methods Compared **eTable 2.** Baseline Characteristics of the Study Cohorts After 1:1 Propensity Score Matching **eTable 3.** Association of ACS Administration With Neonatal Outcomes After 1:1 Propensity Score Matching **eTable 4.** Baseline Characteristics of the Study Cohort (Neonates) **eTable 5.** Association of ACS Administration With Neonatal Outcomes After Overlap Weighting (Neonates) **eFigure 2.** Description of the Study Design for the Analysis of ACS Administration-to-Delivery Intervals **eTable 6.** Baseline Characteristics of the Study Cohorts at Different Intervals of ACS Administration-to-Birth **eTable 7.** Association of ACS Administration With Primary Neonatal Outcomes at Different Intervals of ACS Administration-to-Birth After Adjustment **eTable 8.** Association of ACS Administration With Other Secondary Neonatal Outcomes at Different Intervals of ACS Administration-to-Birth After Adjustment

Supplement 2.
Data Sharing Statement
