# Supplementary material for: Antenatal Corticosteroid Treatment During the Late-Preterm Period and Neonatal Outcomes for Twin Pregnancies
Source: JAMA Netw Open. 2023 Nov 17;6(11):e2343781. doi: 10.1001/jamanetworkopen.2023.43781 (PMC10656637; doi:10.1001/jamanetworkopen.2023.43781)
Supplement: Supplement 1. — eTable 1. Definitions of Variables and Outcome Measures eFigure 1. Methods Compared eTable 2. Baseline Characteristics of the Study Cohorts After 1:1 Propensity Score Matching eTable 3. Association of ACS Administration With Neonatal Outcomes After 1:1 Propensity Score Matching eTable 4. Baseline Characteristics of the Study Cohort (Neonates) eTable 5. Association of ACS Administration With Neonatal Outcomes After Overlap Weighting (Neonates) eFigure 2. Description of the Study Design for the Analysis of ACS Administration-to-Delivery Intervals eTable 6. Baseline Characteristics of the Study Cohorts at Different Intervals of ACS Administration-to-Birth eTable 7. Association of ACS Administration With Primary Neonatal Outcomes at Different Intervals of ACS Administration-to-Birth After Adjustment eTable 8. Association of ACS Administration With Other Secondary Neonatal Outcomes at Different Intervals of ACS Administration-to-Birth After Adjustment [file jamanetwopen-e2343781-s001.pdf]

# Supplementary Online Content

Zhu J, Zhao Y, An P, et al. Antenatal corticosteroid treatment during the late-preterm period and neonatal outcomes for twin pregnancies. *JAMA Netw Open.* 2023;6(11):e2343781. doi:10.1001/jamanetworkopen.2023.43781

**eTable 1.** Definitions of Variables and Outcome Measures
**eFigure 1.** Methods Compared
**eTable 2.** Baseline Characteristics of the Study Cohorts After 1:1 Propensity Score Matching
**eTable 3.** Association of ACS Administration With Neonatal Outcomes After 1:1 Propensity Score Matching
**eTable 4.** Baseline Characteristics of the Study Cohort (Neonates)
**eTable 5.** Association of ACS Administration With Neonatal Outcomes After Overlap Weighting (Neonates)
**eFigure 2.** Description of the Study Design for the Analysis of ACS Administration-to-Delivery Intervals
**eTable 6.** Baseline Characteristics of the Study Cohorts at Different Intervals of ACS Administration-to-Birth
**eTable 7.** Association of ACS Administration With Primary Neonatal Outcomes at Different Intervals of ACS Administration-to-Birth After Adjustment
**eTable 8.** Association of ACS Administration With Other Secondary Neonatal Outcomes at Different Intervals of ACS Administration-to-Birth After Adjustment

This supplementary material has been provided by the authors to give readers additional information about their work.

**eTable 1.** Definitions of Variables and Outcome Measures

| **Variables** | **Definition** |
|---|---|
| Growth discordance | Twin birthweight difference >20%, calculated as: [(birthweight of larger twin - birthweight of smaller twin)/birthweight of larger twin)]x100% |
| Transferred with respiratory complications | Being under poor respiratory conditions such as RDS and transferred to the local children's hospital for further treatment evaluated by neonatologists at delivery |
| Respiratory distress syndrome | The clinical signs of respiratory distress presented as tachypnea, retractions, flaring, grunting, or cyanosis, with a chest x-ray showing hypoaeration and reticulogranular infiltrates at delivery |
| Surfactant use | Administration of surfactant as neonatal respiratory treatment at delivery |
| Asphyxia | Severe respiratory depression at birth, incapable of commencing effective spontaneous breathing by 1 minute or up to 5 minutes after birth; mild asphyxia defined as an Apgar score of less than or equal to 7 for one minute or less than or equal to 7 for five minutes; severe asphyxia as an Apgar score of less than or equal to 3 for one minute or less than or equal to 5 for five minutes |
| Transient tachypnea | Signs of respiratory distress, specifically tachypnea, which are resolved at age of 72 hours |
| Necrotizing enterocolitis | Modified Bell Stage 2 or 3; Stage 2: Clinical signs and symptoms with pneumatosis intestinalis on radiographs, and Stage 3: Advanced clinical signs and symptoms, pneumatosis, impending or proven intestinal perforation |
| Sepsis | Clinical suspicion of systemic infection with positive blood, cerebral spinal fluid, or catheterized/suprapubic urine culture; or, in the absence of positive cultures, clinical evidence of cardiovascular collapse or an X-ray confirming infection |
| Hyperbilirubinemia | The level of total bilirubin greater than the 95$^{th}$ percentile of the corresponding hourly age |
| Hyperglycemia | Whole blood glucose >125 mg/dl (to convert to millimoles per liter, multiply by 0.0555) or plasma glucose >145 mg/dl (to convert to millimoles per liter, multiply by 0.0555) |
| Hypoglycemia | A glucose level of <40 mg per deciliter (to convert to millimoles per liter, multiply by 0.0555) at any time |
| Acidosis | PH <7.20 and/or base deficit < -12.00 mmol/L of umbilical artery |

© 2023 Zhu J et al. *JAMA Network Open.*

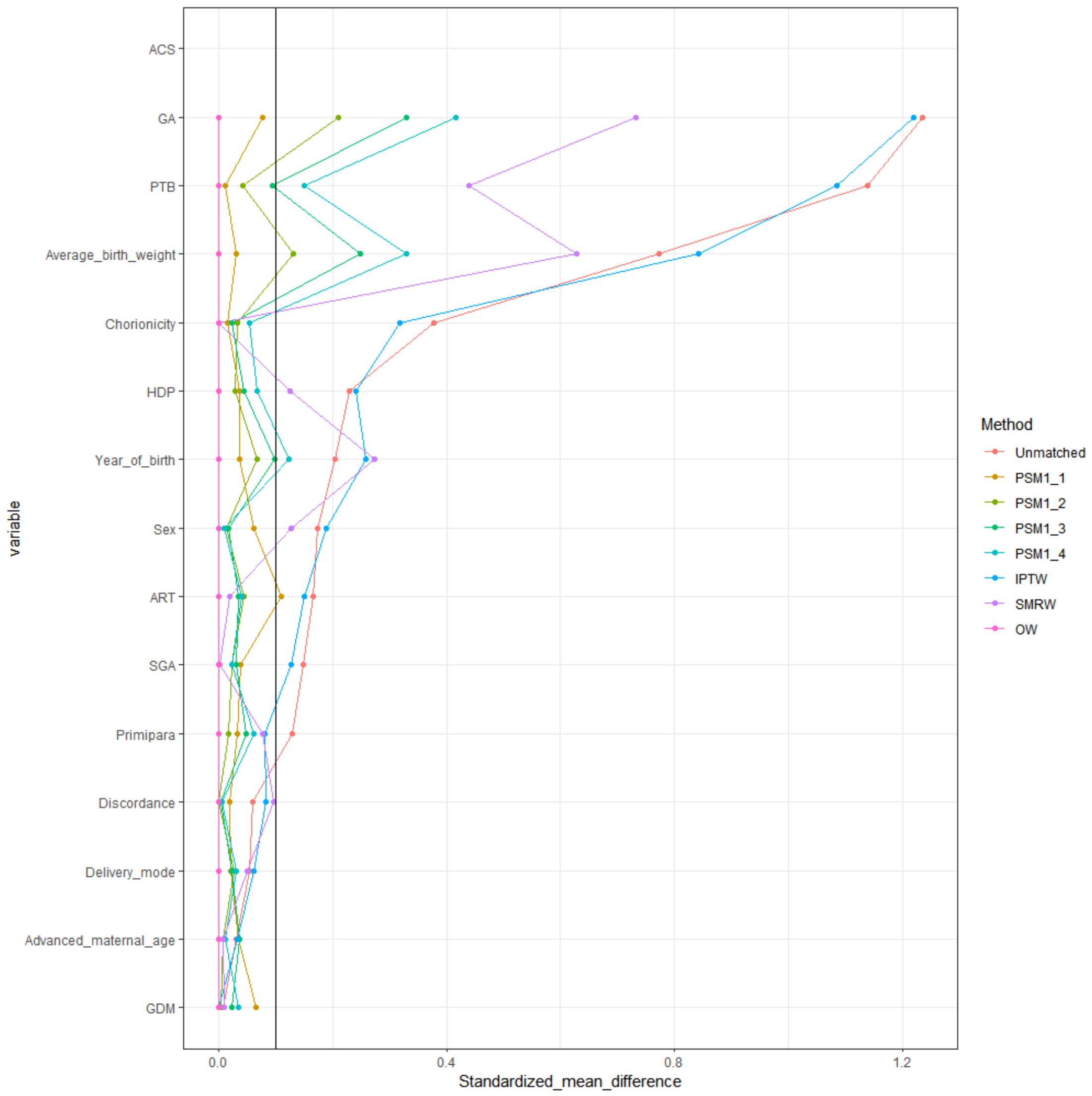


**eFigure 1.** Methods Compared

For IPTW, the inverse propensity score applied as weights for those who received ACS, and the inverse of 1 minus the propensity score for those who did not; for SMRW, 1 applied as weights for those who received ACS, and propensity score multiplied by the inverse of 1 minus the propensity score for those who did not; for overlap weighting, 1 minus the propensity score applied as weights for those who received ACS, and the propensity score for those who did not

Abbreviations: ACS, antenatal corticosteroid; GA, gestational age; PTB, preterm birth; HDP, hypertensive disorders in pregnancy; ART, assisted reproductive technology; SGA, small for gestational age; GDM, gestational diabetes mellitus; PSM, propensity score matching; IPTW, inverse probability of treatment weighting; SMRW, standardized mortality ratio weighting; OW, overlap weighting

© 2023 Zhu J et al. *JAMA Network Open.*

**eTable 2.** Baseline Characteristics of the Study Cohorts After 1:1 Propensity Score Matching

| Characteristic | | No-ACS | ACS | *P* value | Absolute standardized mean difference |
|---|---|---|---|---|---|
| **N.** | | 293 | 293 | | |
| **Maternal characteristics** | | | | | |
| Year of delivery | | | | 0.73 | 0.036 |
| | 2013-2016 | 185(60.1) | 190(64.8) | | |
| | 2017-2020 | 108(36.9) | 103(35.2) | | |
| Maternal age, mean (SD), y | | 30.7(3.8) | 30.8(4.2) | 0.86 | 0.014 |
| Advanced maternal age | | 52(17.7) | 56(19.1) | 0.75 | 0.035 |
| Primipara | | 260(88.7) | 257(87.7) | 0.80 | 0.032 |
| ART | | 150(51.2) | 134(45.7) | 0.22 | 0.109 |
| GD | | 68(23.2) | 60(20.5) | 0.48 | 0.066 |
| HDP | | 103(35.2) | 98(33.4) | 0.73 | 0.036 |
| Gestational age, wk | | | | 0.95 | 0.077 |
| | 34 | 70(23.9) | 68(23.2) | | |
| | 35 | 78(26.6) | 79(27.0) | | |
| | 36 | 114(38.9) | 114(38.9) | | |
| | 37 | 27(9.2) | 30(10.2) | | |
| | 38 | 4(1.4) | 2(0.7) | | |
| Preterm birth | | 262(89.4) | 261(89.1) | >0.99 | 0.011 |
| Indication for preterm birth | | | | 0.34 | 0.189 |
| | Preterm labor with intact membranes | 15(5.7) | 14(5.4) | | |
| | Ruptured membranes | 59(22.5) | 60(23.0) | | |
| | Expected delivery for HDP | 79(30.2) | 86(33.0) | | |
| | Expected delivery for FGR | 14(5.3) | 5(1.9) | | |
| | Expected delivery for other indication [a] | 95(36.3) | 96(36.8) | | |
| Cesarean delivery | | 285(97.3) | 284(96.9) | >0.99 | 0.020 |
| Dichorionicity | | 197(67.2) | 195(66.6) | 0.93 | 0.015 |
| **Neonatal characteristic** | | | | | |
| Birth weight of both twin infants from same mother, mean (SD), g | | 2,399.4(297.1) | 2,389.8(316.7) | 0.71 | 0.031 |
| At least 1 infant with SGA | | 75(25.6) | 80(27.3) | 0.71 | 0.039 |
| Growth discordance | | 42(14.3) | 40(13.7) | 0.91 | 0.020 |
| Same-sex twin | | 215(73.4) | 207(70.6) | 0.52 | 0.061 |

All data presented as n (%) unless otherwise indicated

[a]Other indications: breech presentation, shoulder presentation, placenta accrete, placenta previa, intrahepatic cholestasis of pregnancy, monochorionic twins, or others

© 2023 Zhu J et al. *JAMA Network Open.*

Abbreviations: ACS, antenatal corticosteroid; ART, assisted reproductive technology; GD, gestational diabetes; HDP, hypertensive disorders in pregnancy; FGR, fetal growth restriction; SGA, small for gestational age

© 2023 Zhu J et al. *JAMA Network Open.*

**eTable 3.** Association of ACS Administration With Neonatal Outcomes After 1:1 Propensity Score Matching

| Outcome[a] | No-ACS | ACS | OR(95%CI) | *P* value | aOR(95%CI)[c] | *P* value |
|---|---|---|---|---|---|---|
| **Primary outcome[b]** | 20(6.8) | 24(8.2) | 1.21(0.66-2.28) | 0.53 | 1.23(0.65-2.35) | 0.53 |
| Transferred with respiratory complications | 6(2.0) | 9(3.1) | 1.52(0.54-4.57) | 0.44 | 1.52(0.54-4.58) | 0.44 |
| Mechanical ventilation | 0 | 0 | NA | NA | NA | NA |
| Respiratory distress syndrome | 15(5.1) | 17(5.8) | 1.14(0.56-2.36) | 0.72 | 1.15(0.55-2.43) | 0.72 |
| Surfactant use | 17(5.8) | 18(6.1) | 1.06(0.53-2.12) | 0.86 | 1.06(0.52-2.17) | 0.87 |
| Neonatal death | 0 | 0 | NA | NA | NA | NA |
| **Secondary outcome** | | | | | | |
| CPAP use | 40(13.7) | 43(14.7) | 1.09(0.68-1.74) | 0.72 | 1.10(0.66-1.82) | 0.72 |
| CPAP use for >2d | 13(4.4) | 15(5.1) | 1.16(0.54-2.52) | 0.70 | 1.17(0.53-2.59) | 0.70 |
| Asphyxia | 9(3.1) | 5(1.7) | 0.55(0.17-1.61) | 0.29 | 0.55(0.17-1.60) | 0.29 |
| Severe asphyxia | 2(0.7) | 2(0.7) | 1.00(0.12-8.38) | >0.99 | 1.00(0.12-8.38) | >0.99 |
| Transient tachypnea | 113(38.6) | 122(41.6) | 1.14(0.82-1.58) | 0.45 | 1.17(0.81-1.69) | 0.41 |
| NICU | 211(72.0) | 215(73.4) | 1.07(0.74-1.54) | 0.71 | 1.10(0.72-1.68) | 0.67 |
| NICU for ≥3d | 199(67.9) | 205(70.0) | 1.10(0.78-1.56) | 0.59 | 1.13(0.76-1.69) | 0.54 |
| Necrotizing enterocolitis | 0 | 0 | NA | NA | NA | NA |
| Sepsis | 2(0.7) | 0 | NA | NA | NA | NA |
| Hyperbilirubinemia | 119(40.6) | 90(30.7) | 0.65(0.46-0.91) | 0.01 | 0.63(0.44-0.90) | 0.01 |
| Hyperglycemia | 10(3.4) | 7(2.4) | 0.69(0.25-1.83) | 0.46 | 0.69(0.24-1.83) | 0.45 |
| Hypoglycemia | 26(8.9) | 32(10.9) | 1.26(0.73-2.19) | 0.41 | 1.26(0.73-2.20) | 0.41 |
| Acidosis | 67(22.9) | 65(22.2) | 0.96(0.65-1.42) | 0.84 | 0.95(0.63-1.44) | 0.82 |

[a]Definitions of outcome measures listed in eTable 1

[b]The primary outcome measure was a composite neonatal respiratory morbidity outcome defined as at least one of the following occurrences in at least one neonate of the twins: respiratory distress syndrome, mechanical ventilation, surfactant administration, transferred with respiratory complications, or neonatal death

[c]Adjusted for propensity score

Abbreviations: ACS, antenatal corticosteroid; OR, odds ratio; CI, confidence interval; aOR, adjusted odds ratio; CPAP, continuous positive airway pressure; NICU, neonatal intensive care unit; NA, not applicable

© 2023 Zhu J et al. *JAMA Network Open.*

**eTable 4.** Baseline Characteristics of the Study Cohort (Neonates)

| Characteristic | | No-ACS | ACS | *P* value | Absolute standardized mean difference |
|---|---|---|---|---|---|
| **N** | | 3342 | 606 | | |
| **Maternal characteristics** | | | | | |
| Year of delivery | | | | <0.001 | 0.205 |
| | 2013-2016 | 1862(55.7) | 398(65.7) | | |
| | 2017-2020 | 1480(44.3) | 208(34.3) | | |
| Maternal age, mean (SD), y | | 31.2(4.0) | 30.8(4.2) | 0.01 | 0.108 |
| Advanced maternal age | | 670(20.0) | 114(18.8) | 0.51 | 0.031 |
| Primipara | | 3074(92.0) | 534(88.1) | 0.003 | 0.129 |
| ART | | 1798(53.8) | 276(45.5) | <0.001 | 0.166 |
| GD | | 664(19.9) | 122(20.1) | 0.87 | 0.007 |
| HDP | | 790(23.6) | 206(34.0) | <0.001 | 0.230 |
| Gestational age, wk | | | | <0.001 | 1.236 |
| | 34 | 160(4.8) | 156(25.7) | | |
| | 35 | 354(10.6) | 158(26.1) | | |
| | 36 | 908(27.2) | 228(37.6) | | |
| | 37 | 1630(48.8) | 60(9.9) | | |
| | 38 | 270(8.1) | 4(0.7) | | |
| | 39 | 8(0.2) | 0 | | |
| | 40 | 12(0.4) | 0 | | |
| Preterm birth | | 1422(42.5) | 542(89.4) | <0.001 | 1.139 |
| Indication for preterm birth | | | | 0.001 | 0.219 |
| | Preterm labor with intact membranes | 86(6.0) | 30(5.5) | | |
| | Ruptured membranes | 302(21.2) | 126(23.2) | | |
| | Expected delivery for HDP | 372(26.2) | 180(33.2) | | |
| | Expected delivery for FGR | 60(4.2) | 10(1.8) | | |
| | Expected delivery for other indication [a] | 602(42.3) | 196(36.2) | | |
| Cesarean delivery | | 132(3.9) | 18(3.0) | 0.30 | 0.054 |
| Dichorionicity | | 2758(82.5) | 402(66.3) | <0.001 | 0.378 |
| **Neonatal characteristics** | | | | | |
| Second twin | | 1671(50.0) | 303(50.0) | >0.99 | <0.001 |
| Birth weight, mean (SD), g | | 2610.6(344.5) | 2371.5(374.7) | <0.001 | 0.665 |
| SGA | | 723(21.6) | 103(17.0) | 0.009 | 0.118 |
| Growth discordance | | 412(12.3) | 86(14.2) | 0.21 | 0.055 |
| Female sex | | 1661(49.7) | 273(45.0) | 0.04 | 0.093 |

All data presented as n (%) unless otherwise indicated

[a]Other indications: breech presentation, shoulder presentation, placenta accrete, placenta previa, intrahepatic cholestasis of pregnancy, monochorionic twins, or others

© 2023 Zhu J et al. *JAMA Network Open.*

Abbreviations: ACS, antenatal corticosteroid; ART, assisted reproductive technology; GD, gestational diabetes; HDP, hypertensive disorders in pregnancy; FGR, fetal growth restriction; SGA, small for gestational age

© 2023 Zhu J et al. *JAMA Network Open.*

**eTable 5.** Association of ACS Administration With Neonatal Outcomes After Overlap Weighting (Neonates)

| **Outcome(%)**[a] | **No-ACS** | **ACS** | **OR(95%CI)** | ***P* value** |
|---|---|---|---|---|
| **Primary outcomes**[b] | 4.4 | 5.9 | 1.36(0.74-2.55) | 0.32 |
| Transferred with respiratory complications | 1.7 | 2.4 | 1.40(0.54-3.81) | 0.49 |
| Mechanical ventilation | 0.1 | 0 | NA | NA |
| Respiratory distress syndrome | 3.3 | 4.3 | 1.32(0.65-2.75) | 0.44 |
| Surfactant use | 3.3 | 4.1 | 1.26(0.61-2.62) | 0.54 |
| Neonatal death | 0 | 0 | NA | NA |
| **Secondary outcome** | | | | |
| CPAP use | 9.9 | 10.8 | 1.10(0.70-1.71) | 0.69 |
| CPAP use for >2d | 2.4 | 2.6 | 1.08(0.45-2.61) | 0.87 |
| Asphyxia | 2.2 | 0.8 | 0.34(0.08-1.12) | >0.99 |
| Severe asphyxia | 0.4 | 0.4 | 1.08(0.10-12.30) | 0.95 |
| Transient tachypnea | 26.6 | 28.7 | 1.11(0.82-1.50) | 0.51 |
| NICU | 56.2 | 57.4 | 1.05(0.80-1.38) | 0.73 |
| NICU for ≥3d | 52.1 | 53.4 | 1.05(0.80-1.38) | 0.71 |
| Necrotizing enterocolitis | 0 | 0 | NA | NA |
| Sepsis | 0.5 | 0 | NA | NA |
| Hyperbilirubinemia | 26.9 | 22.5 | 0.79(0.58-1.08) | 0.14 |
| Hyperglycemia | 2.0 | 1.1 | 0.54(0.16-1.63) | 0.29 |
| Hypoglycemia | 4.6 | 6.6 | 1.45(0.80-2.67) | 0.22 |
| Acidosis | 14.0 | 12.3 | 0.86(0.58-1.28) | 0.46 |

[a]Definitions of outcome measures listed in eTable 1

[b]The primary outcome measure was a composite neonatal respiratory morbidity outcome defined as at least one of the following occurrences in at least one neonate of the twins: respiratory distress syndrome, mechanical ventilation, surfactant administration, transferred with respiratory complications, or neonatal death

Abbreviations: ACS, antenatal corticosteroid; OR, odds ratio; CI, confidence interval; CPAP, continuous positive airway pressure; NICU, neonatal intensive care unit; NA, not applicable

© 2023 Zhu J et al. *JAMA Network Open.*

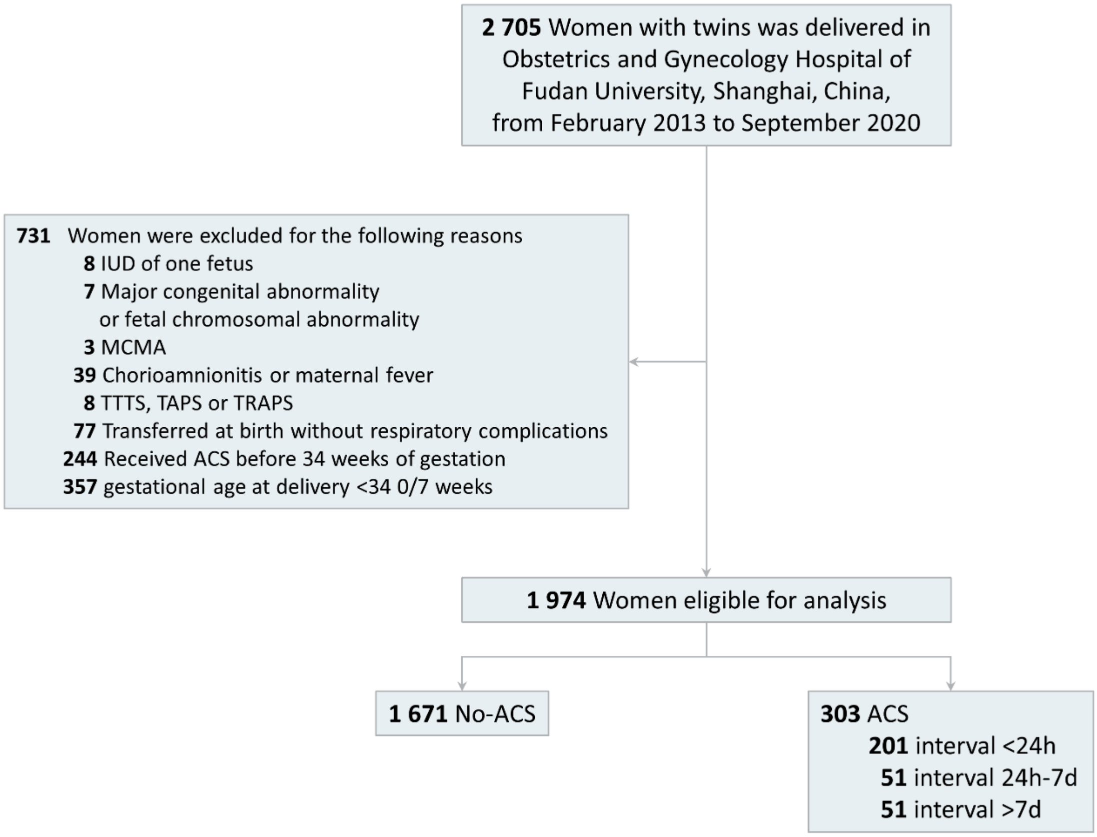


**eFigure 2.** Description of the Study Design for the Analysis of ACS Administration-to-Delivery Intervals

Abbreviations: ACS, antenatal corticosteroid; IUD, intrauterine death; MCMA, monochorionic monoamniotic; TTTS, twin-to-twin transfusion syndrome; TAPS, twin anemia-polycythemia sequence; TRAPS, twin reversed arterial perfusion sequence

© 2023 Zhu J et al. *JAMA Network Open.*

**eTable 6.** Baseline Characteristics of the Study Cohorts at Different Intervals of ACS Administration-to-Birth

| Characteristic | | No-ACS | ACS Administration-to-birth Interval<br><24h | 24h-7d | >7d | *P* value |
|---|---|---|---|---|---|---|
| **N.** | | 1671 | 201 | 51 | 51 | |
| **Maternal characteristic** | | | | | | |
| Year of delivery | | | | | | <0.001 |
| | 2013-2016 | 931(55.7) | 164(81.6) | 16(31.4) | 19(37.3) | |
| | 2017-2020 | 740(44.3) | 37(18.4) | 35(68.6) | 32(62.7) | |
| Maternal age, mean (SD), y | | 31.2(4.0) | 30.5(4.0) | 32.2(4.8) | 30.5(3.9) | 0.01 |
| Advanced maternal age | | 335(20.0) | 35(17.4) | 16(31.4) | 6(11.8) | 0.08 |
| Primipara | | 1537(92.0) | 181(90.0) | 41(80.4) | 45(88.2) | 0.03 |
| ART | | 899(53.8) | 88(43.8) | 25(49.0) | 25(49.0) | 0.05 |
| GD | | 332(19.9) | 44(21.9) | 12(23.5) | 5(9.8) | 0.22 |
| HDP | | 395(23.6) | 64(31.8) | 21(41.2) | 18(35.3) | 0.001 |
| Gestational age, wk | | | | | | <0.001 |
| | 34 | 80(4.8) | 69(34.3) | 9(17.6) | 0(0.0) | |
| | 35 | 177(10.6) | 56(27.9) | 16(31.4) | 7(13.7) | |
| | 36 | 454(27.2) | 76(37.8) | 19(37.3) | 19(37.3) | |
| | 37 | 815(48.8) | 0(0.0) | 7(13.7) | 23(45.1) | |
| | 38 | 135(8.1) | 0(0.0) | 0(0.0) | 2(3.9) | |
| | 39 | 4(0.2) | 0(0.0) | 0(0.0) | 0(0.0) | |
| | 40 | 6(0.4) | 0(0.0) | 0(0.0) | 0(0.0) | |
| Preterm birth | | 711(42.5) | 201(100.0) | 44(86.3) | 26(51.0) | <0.001 |
| Indication for preterm birth | | | | | | <0.001 |
| | Preterm labor with intact membranes | 43(6.0) | 10(5.0) | 3(6.8) | 2(7.7) | |

© 2023 Zhu J et al. *JAMA Network Open.*

| | | | | | |
|---|---|---|---|---|---|
| Ruptured membranes | 151(21.2) | 58(28.9) | 4(9.1) | 1(3.8) | |
| Expected delivery for HDP | 186(26.2) | 61(30.3) | 19(43.2) | 10(38.5) | |
| Expected delivery for FGR | 30(4.2) | 2(1.0) | 3(6.8) | 0(0.0) | |
| Expected delivery for other indication [a] | 301(42.3) | 70(34.8) | 15(34.1) | 13(50.0) | |
| Cesarean delivery | 1605(96.1) | 197(98.0) | 48(94.1) | 49(96.1) | 0.38 |
| Dichorionicity | 1379(82.5) | 130(64.7) | 31(60.8) | 40(78.4) | <0.001 |
| **Neonatal characteristics** | | | | | |
| Birth weight of both twin infants from same mother, mean (SD), g | 2,610.6(289.8) | 2,326.9(315.4) | 2,324.9(338.2) | 2,593.5(279.9) | <0.001 |
| At least 1 infant with SGA | 589(35.2) | 49(24.4) | 19(37.3) | 18(35.3) | 0.02 |
| Growth discordance | 203(12.1) | 31(15.4) | 6(11.8) | 6(11.8) | 0.60 |
| Same-sex twin | 1056(63.2) | 146(72.6) | 39(76.5) | 31(60.8) | 0.01 |
| **Interval, d** | NA | 0 | 2.67(1.66) | 15.49(5.62) | <0.001 |

All data presented as n (%) unless otherwise indicated

[a]Other indication: breech presentation, shoulder presentation, placenta accrete, placenta previa, intrahepatic cholestasis of pregnancy, monochorionic twins, or others

Abbreviations: ACS, antenatal corticosteroid; ART, assisted reproductive technology; GD, gestational diabetes; HDP, hypertensive disorders in pregnancy; FGR, fetal growth restriction; SGA, small for gestational age; NA, not applicable

© 2023 Zhu J et al. *JAMA Network Open.*

**eTable 7.** Association of ACS Administration With Primary Neonatal Outcomes at Different Intervals of ACS Administration-to-Birth After Adjustment

| Outcome[a] | | Rate/N(%) | aOR(95%CI)[c] | *P* value |
|---|---|---|---|---|
| **Primary outcomes[b]** | | | | |
| | **No-ACS** | 41(2.5) | reference | - |
| | **<24h** | 25(12.4) | 1.48(0.79-2.73) | 0.21 |
| | **24h-7d** | 3(5.9) | 0.92(0.43-1.65) | 0.80 |
| | **>7d** | 1(2.0) | 0.96(0.36-1.70) | 0.90 |
| **Transferred with respiratory complications** | | | | |
| | **No-ACS** | 16(1.0) | reference | - |
| | **<24h** | 9(4.5) | 1.76(0.66-4.51) | 0.25 |
| | **24h-7d** | 2(3.9) | 1.44(0.54-2.99) | 0.38 |
| | **>7d** | 1(2.0) | 1.32(0.49-2.43) | 0.46 |
| **Mechanical ventilation** | | | | |
| | **No-ACS** | 4(0.2) | reference | - |
| | **<24h** | 0(0.0) | NA | NA |
| | **24h-7d** | 0(0.0) | NA | NA |
| | **>7d** | 0(0.0) | NA | NA |
| **Respiratory distress syndrome** | | | | |
| | **No-ACS** | 25(1.5) | reference | - |
| | **<24h** | 20(10.0) | 1.55(0.76-3.15) | 0.23 |
| | **24h-7d** | 1(2.0) | 0.57(0.13-1.35) | 0.30 |
| | **>7d** | 0(0.0) | NA | NA |
| **Surfactant use** | | | | |
| | **No-ACS** | 28(1.7) | reference | - |
| | **<24h** | 20(10.0) | 1.49(0.74-2.96) | 0.26 |
| | **24h-7d** | 1(2.0) | 0.55(0.13-1.30) | 0.27 |
| | **>7d** | 0(0.0) | NA | NA |
| **Neonatal death** | | | | |
| | **No-ACS** | 1(0.1) | reference | - |
| | **<24h** | 0(0.0) | NA | NA |
| | **24h-7d** | 0(0.0) | NA | NA |
| | **>7d** | 0(0.0) | NA | NA |

[a]Definitions of outcome measures listed in eTable 1

[b]The primary outcome measure was a composite neonatal respiratory morbidity outcome defined as at least one of the following occurrences in at least one neonate of the twins: respiratory distress syndrome, mechanical ventilation, surfactant administration, transferred with respiratory complications, or neonatal death

[c]Adjusted for year of delivery, advanced maternal age, primipara, gestational age, delivery mode, assisted reproductive technology, chorionicity, preterm birth, gestational diabetes, hypertensive disorders in pregnancy, birth weight of both twin infants from same mother, at least 1 infant with small for gestational age, intertwin growth discordance, and infant sex

© 2023 Zhu J et al. *JAMA Network Open.*

Abbreviations: ACS, antenatal corticosteroid; aOR, adjusted odds ratio; CI, confidence interval; NA, not applicable

© 2023 Zhu J et al. *JAMA Network Open.*

**eTable 8.** Association of ACS Administration With Other Secondary Neonatal Outcomes at Different Intervals of ACS Administration-to-Birth After Adjustment

| Outcome[a] | | Rate/N(%) | aOR(95%CI)[b] | *P* value |
|---|---|---|---|---|
| **CPAP use** | | | | |
| | **No-ACS** | 93(5.6) | reference | - |
| | **<24h** | 38(18.9) | 1.03(0.59-1.76) | 0.92 |
| | **24h-7d** | 10(19.6) | 0.88(0.55-1.36) | 0.58 |
| | **>7d** | 1(2.0) | 0.59(0.22-1.05) | 0.14 |
| **CPAP use for >2d** | | | | |
| | **No-ACS** | 26(1.6) | reference | - |
| | **<24h** | 10(5.0) | 0.93(0.36-2.32) | 0.89 |
| | **24h-7d** | 4(7.8) | 1.05(0.51-1.93) | 0.88 |
| | **>7d** | 1(2.0) | 1.04(0.38-1.91) | 0.92 |
| **Asphyxia** | | | | |
| | **No-ACS** | 51(3.1) | reference | - |
| | **<24h** | 5(2.5) | 0.65(0.21-1.68) | 0.42 |
| | **24h-7d** | 1(2.0) | 0.70(0.16-1.58) | 0.50 |
| | **>7d** | 0(0.0) | NA | NA |
| **Severe Asphyxia** | | | | |
| | **No-ACS** | 12(0.7) | reference | - |
| | **<24h** | 3(1.5) | 1.94(0.35-9.54) | 0.41 |
| | **24h-7d** | 0(0.0) | NA | NA |
| | **>7d** | 0(0.0) | NA | NA |
| **Transient tachypnea** | | | | |
| | **No-ACS** | 338(20.2) | reference | - |
| | **<24h** | 103(51.2) | 1.25(0.86-1.83) | 0.25 |
| | **24h-7d** | 17(33.3) | 0.77(0.54-1.08) | 0.14 |
| | **>7d** | 9(17.6) | 0.90(0.67-1.18) | 0.48 |
| **NICU** | | | | |
| | **No-ACS** | 805(48.2) | reference | - |
| | **<24h** | 162(80.6) | 1.33(0.81-2.21) | 0.27 |
| | **24h-7d** | 40(78.4) | 1.09(0.69-1.78) | 0.71 |
| | **>7d** | 23(45.1) | 0.88(0.68-1.12) | 0.31 |
| **NICU for ≥3d** | | | | |
| | **No-ACS** | 763(45.7) | reference | - |
| | **<24h** | 152(75.6) | 1.16(0.73-1.84) | 0.54 |
| | **24h-7d** | 38(74.5) | 1.05(0.68-1.66) | 0.82 |
| | **>7d** | 22(43.1) | 0.90(0.71-1.15) | 0.42 |
| **Necrotizing enterocolitis** | | | | |
| | **No-ACS** | 1(0.1) | reference | - |
| | **<24h** | 0(0.0) | NA | NA |
| | **24h-7d** | 0(0.0) | NA | NA |
| | **>7d** | 0(0.0) | NA | NA |
| **Sepsis** | | | | |

© 2023 Zhu J et al. *JAMA Network Open.*

| | | | | |
|---|---|---|---|---|
| | **No-ACS** | 6(0.4) | reference | - |
| | **<24h** | 0(0.0) | NA | NA |
| | **24h-7d** | 0(0.0) | NA | NA |
| | **>7d** | 0(0.0) | NA | NA |
| **Hyperbilirubinemia** | | | | |
| | **No-ACS** | 397(23.8) | reference | - |
| | **<24h** | 59(29.4) | 0.60(0.39-0.90) | 0.02 |
| | **24h-7d** | 23(45.1) | 0.83(0.59-1.16) | 0.28 |
| | **>7d** | 13(25.5) | 0.92(0.71-1.16) | 0.49 |
| **Hyperglycemia** | | | | |
| | **No-ACS** | 24(1.4) | reference | - |
| | **<24h** | 7(3.5) | 0.99(0.34-2.56) | 0.98 |
| | **24h-7d** | 0(0.0) | NA | NA |
| | **>7d** | 0(0.0) | NA | NA |
| **Hypoglycemia** | | | | |
| | **No-ACS** | 69(4.1) | reference | - |
| | **<24h** | 16(8.0) | 1.19(0.59-2.30) | 0.61 |
| | **24h-7d** | 13(25.5) | 1.92(1.29-2.81) | <0.001 |
| | **>7d** | 4(7.8) | 1.19(0.76-1.69) | 0.39 |
| **Acidosis** | | | | |
| | **No-ACS** | 184(11.0) | reference | - |
| | **<24h** | 54(26.9) | 1.02(0.66-1.55) | 0.95 |
| | **24h-7d** | 13(25.5) | 0.99(0.68-1.41) | 0.96 |
| | **>7d** | 1(2.0) | 0.49(0.49-0.84) | 0.04 |

[a]Definitions of outcome measures listed in eTable 1

[b]Adjusted for year of delivery, advanced maternal age, primipara, gestational age, delivery mode, assisted reproductive technology, chorionicity, preterm birth, gestational diabetes, hypertensive disorders in pregnancy, birth weight of both twin infants from same mother, at least 1 infant with small for gestational age, intertwin growth discordance, and infant sex

Abbreviations: ACS, antenatal corticosteroid; aOR, adjusted odds ratio; CI, confidence interval; CPAP, continuous positive airway pressure; NICU, neonatal intensive care unit; NA, not applicable

© 2023 Zhu J et al. *JAMA Network Open.*
