# Supplementary material for: Antenatal Corticosteroid Treatment During the Late-Preterm Period and Neonatal Outcomes for Twin Pregnancies
Source: JAMA Netw Open. 2023 Nov 17;6(11):e2343781. doi: 10.1001/jamanetworkopen.2023.43781 (PMC10656637; doi:10.1001/jamanetworkopen.2023.43781)
Supplement: Supplement 2. — Data Sharing Statement [file jamanetwopen-e2343781-s002.pdf]

Data Sharing Statement

Zhu. Antenatal Corticosteroid Treatment During the Late-Preterm Period and Neonatal Outcomes for Twin Pregnancies. *JAMA Netw Open*. Published November 17, 2023. doi:10.1001/jamanetworkopen.2023.43781

Data
Data available: No
